# Hematoma and Gaseous Gangrene

**Published:** 1917-12

**Authors:** 


***Hematoma and Gaseous Gangrene.*** A Paper Read at the Medical Meeting of the IVth Army Corps in July, 1916. By M. HEITZ-BOYER. Trans. and abs. from the *Archives de Médecine et de Pharmacie Militaires*, November, 1916.

H.-B. calls attention to the relationship that may exist between a localized hematoma and gaseous gangrene in the region just below it. Some such hematomas caused by lesions of the large blood vessels, serve to check a serious hemorrhage, but in so-doing they may lead to gaseous gangrene. This form of gangrene finds favorable conditions in necrotic tissues such as almost surely result from rupture of important vascular trunks, not only because the supply of blood from this source is cut off, but also because the pressure from the hematoma formed tends to interrupt the reestablishment of the collateral circulation. Thus a kind of secondary gaseous gangrene is almost inevitable in the case of soldiers whose clothing and skin present a rich and varied bacterial flora. Often many days after the original wound latent bacteria in such devitalized tissue may show extreme virulence. H.-B. mentions a case of ligature necessitated by a secondary hemorrhage of the forearm, which, in a few hours, under the very eyes of the physician, developed gaseous gangrene.

After referring to Sacquépée's bacteriological study of such phenomena, H.-B. concludes that only amputation can save a case in which this form of gaseous gangrene has set in : but he insists that by proper preventive treatment it may be avoided. In every wound involving lesions of the great blood vessels, he would have the surgeon hunt out carefully all hematomas, open them, and ligature the bleeding vessel.

M. Sacquépée, in the *discussion* of the foregoing contribution, described a case in which the two lower limbs had been crushed by falling earth. On the right limb there were also deep wounds, but on the left limb no surface injuries at all. Gaseous gangrene developed in the wound of the right limb and appeared shortly afterwards in the left limb as well. At the autopsy it was found that large hematomas had formed in the left limb, and that as a result of these, gaseous gangrene, the organism of which had probably been communicated through the blood, had appeared secondarily in that limb also. S. believed, however, that hematoma favors this infection by other means than its mechanical action in stopping the blood supply, for it probably in itself affords an excellent medium in which the latent bacilli are stimulated to great activity. S. declared that the results of experiments made by him on rabbits confirmed this belief. He had inoculated one rabbit with B. bellonensis (Bacillus of malignant gaseous edema) without any previous preparation of the limb. No appreciable result followed. When however he repeated the experiment, first impeding the circulation with a tightly-applied tourniquet, fulminating gaseous gangrene developed. Similarly after producing hematoma in the thigh of another rabbit and inoculating it with the organism, S. found that gaseous formation began in the clot and then spread to all parts of the thigh. S. emphasized, in conclusion, the varying nature of the activities of B. bellonensis, for besides the common edematous variety of gangrene produced by it, various other forms are to be noted; such as, " extended muscular alterations, marked gaseous infiltrations, and modified edema ", due, as it appears, to organisms of relatively feeble virulence.

